# Associations of computer gaming with incident dementia, cognitive functions, and brain structure: a prospective cohort study and Mendelian randomization analysis

**DOI:** 10.1186/s13195-024-01496-7

**Published:** 2024-06-19

**Authors:** Yiming Jia, Mengyao Shi, Pinni Yang, Ruirui Wang, Lulu Sun, Yinan Wang, Qingyun Xu, Jing Zhang, Qilu Zhang, Daoxia Guo, Xiaowei Zheng, Yi Liu, Xinyue Chang, Yu He, Li Hui, Guo-Chong Chen, Yonghong Zhang, Zhengbao Zhu

**Affiliations:** 1 Department of Epidemiology, School of Public Health, Jiangsu Key Laboratory of Preventive and Translational Medicine for Geriatric Diseases, MOE Key Laboratory of Geriatric Diseases and Immunology, Suzhou Medical College of Soochow University, 199 Renai Road, Industrial Park District, Suzhou, Jiangsu Province 215123 China; 2 School of Nursing, Suzhou Medical College of Soochow University, Suzhou, Jiangsu Province 215006 China; 3 Department of Public Health and Preventive Medicine, Wuxi School of Medicine, Jiangnan University, Wuxi, Jiangsu Province 214122 China; 4 Research Center of Biological Psychiatry, The Affiliated Guangji Hospital of Soochow University, Suzhou, Jiangsu Province 215003 China; 5 Department of Nutrition and Food Hygiene, School of Public Health, Suzhou Medical College of Soochow University, Suzhou, Jiangsu Province 215123 China

**Keywords:** Computing gaming, Dementia, Cognitive function, Brain structure, Risk factor

## Abstract

**Background:**

Computer gaming has recently been suggested to be associated with benefits for cognition, but its impact on incident dementia remains uncertain. We aimed to investigate the observational associations of playing computer games with incident dementia, cognitive functions, and brain structural measures, and further explore the genetic associations between computer gaming and dementia.

**Methods:**

We included 471,346 White British participants without dementia at baseline based on the UK Biobank, and followed them until November 2022. We estimated the risk of dementia using Cox proportional hazard models, and assessed the changes of cognitive functions and brain structural measures using logistic regression models and linear regression models. Mendelian randomization (MR) analyses were performed to examine the association between genetically determined computer gaming and dementia.

**Results:**

High frequency of playing computer games was associated with decreased risk of incident dementia (HR, 0.81 [95% CI: 0.69, 0.94]). Individuals with high frequency of playing computer games had better performance in prospective memory (OR, 1.46 [1.26, 1.70]), reaction time (beta, -0.195 [-0.243, -0.147]), fluid intelligence (0.334 [0.286, 0.382]), numeric memory (0.107 [0.047, 0.166]), incorrect pairs matching (-0.253 [-0.302, -0.203]), and high volume of gray matter in hippocampus (0.078 [0.023, 0.134]). Genetically determined high frequency of playing computer games was associated with a low risk of dementia (OR, 0.37 [0.15, 0.91]).

**Conclusions:**

Computer gaming was associated with a decreased risk of dementia, favorable cognitive function, and better brain structure, suggesting that computer gaming could modulate cognitive function and may be a promising target for dementia prevention.

**Supplementary Information:**

The online version contains supplementary material available at 10.1186/s13195-024-01496-7.

## Background

Dementia is a chronic syndrome characterized by progressive cognitive deficits, behavioral impairment, and decline in daily function [1]. In 2019, the global prevalence of dementia was up to 57.4 million, and the number was expected to triple to 152.8 million by 2050 [2]. Given the substantial public health and socioeconomic burden of dementia, better mapping the contributing risk factors of dementia is important for dementia prevention, management, and surveillance.

Computer gaming (also known as video gaming), a common leisure activity involving multiple perceptual and attentional demands, has gained attention for its potential in improving brain structure and enhancing cognition. Accumulative evidence demonstrated that training on 3D-platform games could increase the volume of hippocampus or the functionally connected entorhinal cortex [3], while dance video games had a facilitating effect on prefrontal cortex activity [4]. In recent observational studies, video gamers were reported to have better performance in action cascading and working memory than non-gamers [5, 6]. Several randomized controlled trials (RCTs) also supported the beneficial role of video gaming in mitigating cognitive deterioration [7–10]. Moreover, according to the Mini-Mental State Examination score, video game-based interventions were shown to be able to improve cognitive abilities among patients with mild cognitive impairment or dementia [11]. However, to date, little is known about the impact of computer gaming on incident dementia. Therefore, well-designed prospective studies on the association of computer gaming with dementia are needed. In addition, these existing studies for brain structure and cognition had relative small sample sizes with a limited statistical power, so it is also important to further validate the effect of computer gaming on cognition and brain structure in studies with larger sample size.

Mendelian randomization (MR) is an emerging method using genetic variants associated with exposures as instruments to assess the causality for the associations between exposures and outcomes [12]. Given the random allocation of genetic variants at conception, MR estimates are not influenced by confounding and reverse causation biases in such ‘natural’ RCT [13]. Recently, several risk loci for the frequency of playing computer games has been identified [14, 15], and large genome-wide association study (GWAS) for dementia has been undertaken [16], providing opportunities for making causal inference for the associations between computer gaming and incident dementia using the MR approach.

**Fig. 1:**
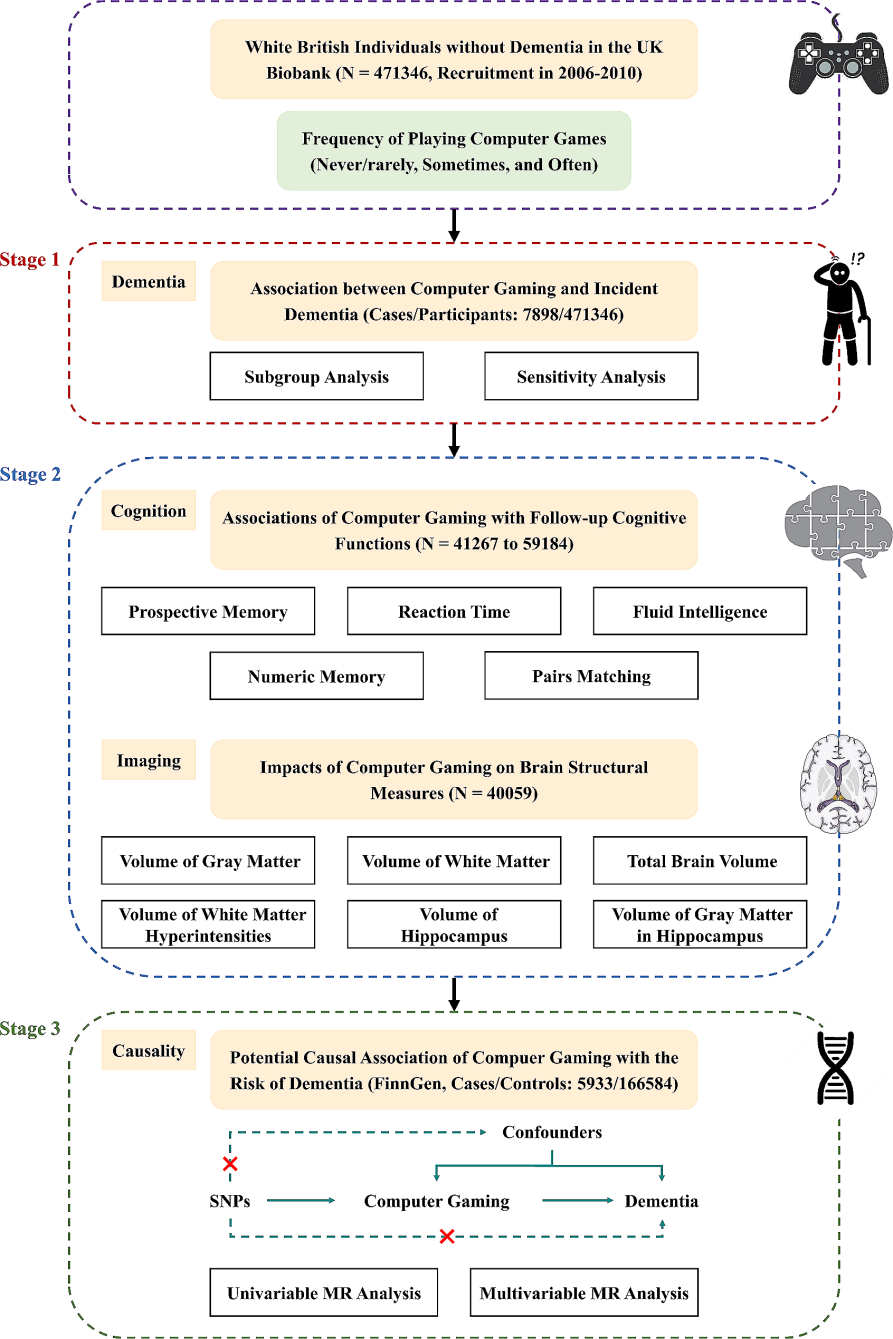
Conceptual workflow of this study. Abbreviations: MR, Mendelian randomization; SNP, single nucleotide polymorphism

Herein, we performed a three-stage study to examine the associations of computer gaming with dementia, cognitive functions, and brain structure (Fig. 1). First, we investigated the observational associations between the frequency of playing computer games and incident dementia in a large prospective study based on the UK Biobank. Second, we further explored the associations of the frequency of playing computer games with 5 cognitive functions and 6 brain structural measures in the prospective study based on the UK Biobank. Third, we conducted a two-sample MR study to evaluate the potential causal association between computer gaming and the risk of dementia among European individuals.

## Methods

### Study population

The UK Biobank is a large prospective observational study established to provide a resource for investigation of the genetic, environmental, and lifestyle factors associated with a wide range of diseases, including dementia [17]. In brief, this study enrolled more than 500,000 ethnically diverse men and women aged 40–69 years who attended one of 22 assessment centers in England, Wales, and Scotland between 2006 and 2010. At recruitment, participants provided a wide range of information on health and diseases and underwent various measurements. The UK Biobank was approved by the National Information Governance Board for Health and Social Care and the National Health Service North West Multicenter Research Ethics Committee (reference 11/NW/0382), and written informed consent was obtained from all participants. The UK Biobank approved the study application (Project ID 91185).

### Computer gaming

Frequency of playing computer games was assessed using a questionnaire generated by the Adaptive Communication Environment system. In this touchscreen questionnaire, participants were asked “Do you play computer games?”, and they were asked to select the corresponding frequency (never/rarely, sometimes, and often) for playing computer games during the past year.

### Dementia assessment

The outcome of interest in stage 1 was incident dementia. The outcome events were detected using hospital inpatient records from the Hospital Episode Statistics for England, Scottish Morbidity Record data for Scotland, and the Patients Episode Database for Wales. Incident dementia was defined by ICD-9 and ICD-10 codes (Table S1). Information on the date of diagnosis was collected through cumulative medical records of hospital diagnoses. Person-time of follow-up was calculated from the date of enrollment through date of diagnosis, death or withdrawal from the study, or end of the most recent follow-up (November 2, 2022), whichever came first.

### Cognition and brain structure assessment

The outcomes of interest in stage 2 included 5 cognitive functions (i.e. prospective memory, reaction time, fluid intelligence, numeric memory, and incorrect pairs matching), which were tested via a touchscreen interface in the UK Biobank assessment center at a single time-point of the imaging visit during 2014 to 2019 [18]. Briefly, these five tests capture the prospective memory, processing speed, verbal and numerical reasoning, attention/working memory, and visuospatial memory of the participants, respectively. A description of the five cognitive tests is listed in the Table S2.

Apart from cognitive functions, 6 brain structural measures (i.e. volume of gray matter, volume of white matter, total brain volume, volume of white matter hyperintensities, volume of hippocampus, and volume of gray matter in hippocampus) obtained from magnetic resonance imaging (MRI) at a single time-point since 2014 (≥ 4.3 years after computer gaming assessment, average time interval = 9.0 years) were also included as the outcomes in stage 2 [19]. Our study used imaging-derived phenotypes generated by an image-processing pipeline developed and conducted on behalf of the UK Biobank. The volumes (in mm^3^) of gray matter, white matter, whole brain, hippocampus, and grey matter in hippocampus were assessed based on T1-weighted structural brain MRI, and the volume of white matter hyperintensities was assessed based on T2-weighted brain MRI. The external surface of the skull was assessed based on T1-weighted MRI and used to normalize brain tissue volumes for head size.

### Covariates

Information on demographic and socioeconomic factors, lifestyle behaviors, and medical history was collected at baseline by a touchscreen questionnaire and nurse-led interviews. The Townsend deprivation index was generated based on 4 socioeconomic variables (unemployment, non-car ownership, non-home ownership, and household overcrowding). Educational attainment was categorized into college/university degree or not. Employment status was categorized into in paid employment/self-employed or other employment status. Diet score was calculated based on the following factors: vegetable intake at least four tablespoons each day; fruit intake at least three pieces each day; fish intake at least twice each week; unprocessed red meat intake no more than twice each week; and processed meat intake no more than twice each week [20]. Each one point was given for each of the aforementioned diet factor, with the total diet score ranging from 0 to 5. Sedentary duration was quantified by summing up the time spent on television watching, using a computer, and driving in every 24 h, and daily sedentary duration was categorized into 4 categories: <4 h, 4 to < 6 h, 6 to < 8 h, and ≥ 8 h. Physical activity was estimated using the International Physical Activity Questionnaire, and participants were categorized into low, medium, and high groups by metabolic equivalent minutes per week. Sleep duration was reported as the hours of sleep in every 24 h (including naps), and daily sleep duration was categorized as short (6 h or less), recommended (7–8 h), and prolonged (9 h or more). Loneliness was assessed by asking two questions: “Do you often feel lonely?” (no, 0; yes, 1) and “How often are you able to confide in someone close to you?” (0, almost daily–once every few months; 1, never or almost never) [21]. Individuals responding positively to both of these two questions were defined as lonely. Medical history, including history of diabetes and history of cardiovascular disease were evaluated based on a combination of ICD-10 codes and self-reported diagnoses.

### Statistical analysis

Among the 471,896 White British participants who consented, we excluded participants who had dementia at baseline (*n* = 307) and those without data on the frequency of playing computer games (*n* = 243). Finally, a total of 471,346 White British participants remained for the main analysis (Figure S1).

Baseline characteristics were summarized by incident dementia status, and data were reported as percentages for categorical variables and as means ± standard deviations for continuous variables. In stage 1, multivariate Cox proportional hazards models were used to estimate hazard ratios (HRs) and 95% confidence intervals (CIs) of incident dementia across the frequency of playing computer games, using the low frequency group that never/rarely played computer games as the reference group. The adjusted covariates including sex, age, age-square, smoking status, alcohol drinking, educational attainment, Townsend deprivation index (in quintile), employment status, diet score, sedentary duration, physical activity, sleep duration, loneliness, history of diabetes, and history of cardiovascular disease.

Subsequently, we conducted subgroup analyses to assess the potential effect modification by several pre-specified dementia-related risk factors. Interactions between the frequency of playing computer games and subgroup variables on incident dementia were tested by the likelihood ratio test, adjusting for the aforementioned covariates unless the variable was used as a subgroup variable. Moreover, we conducted a series of sensitivity analyses to test the robustness of our findings: (1) we further adjusted for baseline frailty status; (2) we further adjusted for baseline social isolation; (3) we further adjusted for baseline depressive symptoms; (4) we further adjusted for baseline medication burden; (5) we further adjusted for family history of dementia; (6) we further adjusted for *APOE* ε4 carrier; (7) we excluded participants with addiction history; and (8) we excluded dementia cases occurring in the first two years of follow-up. Frailty status [22], social isolation [21], depressive symptoms [23], medication burden [24], and *APOE* ε4 carrier [25] were defined based on the established criteria according to previous studies. In addition, we adjusted for each baseline cognitive function in turn to eliminate the impacts of these cognitive functions on the association between computer gaming and dementia.

In stage 2, we used multivariate logistic regression models (for prospective memory) and linear regression models (for reaction time, fluid intelligence, numeric memory, incorrect pairs matching, volume of gray matter, volume of white matter, total brain volume, volume of white matter hyperintensities, volume of hippocampus, and volume of gray matter in hippocampus) to prospectively assess the associations of computer gaming with cognitive functions and brain structure when appropriate. Each cognitive function (except for prospective memory) and brain structural measure was standardized for easier comparison across measures. A 2-tailed *P* value < 0.05 was considered to be statistically significant. All analyses were performed using SAS statistical software (version 9.4; Cary, NC).

### MR analysis

The detailed information concerning MR study design, data sources, genetic instrument selection, and statistical analysis is available in **Supplementary Methods**. In stage 3, we examined the association between genetically determined frequency of playing computer games and the risk of incident dementia via a MR design based on summary-level data with single nucleotide polymorphisms (SNPs) as genetic instruments (Fig. 1). Summary-level data on the frequency of playing computer games was derived from a large-scale GWAS based on the UK Biobank, involving 462,433 European-descent participants [14, 15]. Summary statistics for dementia was obtained from a nationwide Finnish GWAS meta-analysis of 13 cohorts and biobanks with 172,517 participants of European ancestry [16]. The random-effect inverse-variance weighted (IVW) method was used in the main MR analyses [26], and the penalized IVW method [27], the weighted median method [28], the maximum likelihood method [14], the MR-Robust Adjusted Profile Scoring (MR-RAPS) method [29], the MR Pleiotropy Residual Sum and Outlier (MR-PRESSO) method [30], the MR-Egger regression method [31], and the leave-one-out method were used in the sensitivity MR analyses [32]. Moreover, we also performed multivariable MR analyses with adjustment for hypertension, diabetes, hyperlipidemia, smoking, and alcohol drinking to eliminate the influences of these confounders [31]. The MR results were presented as odds ratios (ORs) and their 95% CIs for dementia. All MR analyses were performed in R software (version 3.4.3; R Development Core Team) with ‘gtx’, ‘MendelianRandomization’, ‘MRPRESSO’, and ‘TwoSampleMR’ packages.

## Results

### Baseline characteristics

Baseline characteristics of the participants are presented in Table 1. Among the 471,346 participants included in this study, 372,522 participants (79.0%) never/rarely played computer games, 82,937 participants (17.6%) sometimes played computer games, and 15,887 participants (3.4%) often played computer games. The mean age of the participants was 56.8 years, and 45.5% of the participants were men.

**Table 1:** Characteristics of study participants at baseline

Characteristics	Overall	No dementia (*N* = 463,448)	Incident dementia (*N* = 7898)
Age (years), mean ± SD	56.8 ± 8.0	56.6 ± 8.0	63.9 ± 5.2
Male sex, %	45.5	45.4	53.5
Smoking status, %			
Current	10.5	10.5	12.1
Past	35.6	35.4	43.3
Never	54.0	54.1	44.7
Alcohol drinking, %			
Daily or almost daily	21.1	21.0	22.0
3–4 times a week	23.8	23.9	18.8
Once or twice a week	26.3	26.4	22.4
1–3 times a month	11.2	11.2	10.2
Special occasions only	10.9	10.8	13.7
Never	6.8	6.6	13.0
College or above, %	32.3	32.5	20.5
In paid employment or self-employed, %	57.2	57.8	20.8
Townsend deprivation index, %			
Q1	20.1	20.2	17.4
Q2-Q4	59.9	60.0	56.3
Q5	20.0	19.9	26.3
Diet score, %^*^			
0	0.9	0.8	0.9
1	9.1	9.1	8.4
2	23.8	23.8	23.5
3	33.2	33.2	33.9
4	27.4	27.4	26.9
5	5.7	5.7	6.4
Physical activity, %			
High	40.5	40.5	37.8
Medium	40.8	40.8	41.3
Low	18.7	18.7	20.9
Daily sedentary time, %			
<4 h	37.0	37.1	30.4
4 to < 6 h	34.8	34.8	34.6
6 to < 8 h	17.5	17.4	20.7
≥8 h	10.7	10.6	14.3
Daily sleep duration, %			
6 h or less	24.1	24.0	26.9
7–8 h	68.3	68.4	60.4
9 h or more	7.7	7.6	12.7
Feeling lonely, %	4.8	4.7	7.1
History of diabetes, %	5.0	4.8	14.1
History of cardiovascular disease, %^†^	31.1	30.7	53.5

^*^ Each one point was given for each favorable diet factor (vegetable intake at least four tablespoons each day; fruit intake at least three pieces each day; fish intake at least twice each week; unprocessed red meat intake no more than twice each week; and processed meat intake no more than twice each week), with the total diet score ranging from 0 to 5

^†^ Cardiovascular disease included hypertension, coronary artery disease, stroke, heart failure, atrial fibrillation, and angina

### Computer gaming and incident dementia

During a median 13.7 years (6,261,671 person-years) of follow-up, there were 7898 cases of incident dementia. Compared with participants who never played computer games, the adjusted HRs of dementia were 0.90 (95% CI: 0.83, 0.97) for those who sometimes played computer games and 0.81 (95% CI: 0.69, 0.94) for those who often played computer games (*P* for trend < 0.001) (Table 2).

**Table 2:** Association between computer gaming and incident dementia

	Frequency of playing computer games			*P* for trend
	Never/rarely (*N* = 372,522)	Sometimes (*N* = 82,937)	Often (*N* = 15,887)	
**All-cause dementia**				
No. of cases	6482	1170	246	
Model 1 (HR [95% CI])	1.00 (reference)	0.91 (0.84, 0.98)	0.82 (0.70, 0.96)	< 0.001
Model 2 (HR [95% CI])	1.00 (reference)	0.90 (0.83, 0.97)	0.81 (0.69, 0.94)	< 0.001

Model 1 was adjusted for sex, age, age-square, smoking status, alcohol drinking, educational attainment, Townsend deprivation index, employment status, diet score, sedentary duration, physical activity, sleep duration, and loneliness

Model 2 was based on model 1 and additionally adjusted for history of diabetes and history of cardiovascular disease

In the subgroup analyses, no significant interaction between the frequency of playing computer games and the pre-specified factors was observed (all *P* for interaction > 0.05), and high frequency of playing computer games was significantly associated with a decreased risk of dementia in most subgroups (Table S3). In the sensitivity analyses, the association between playing computer games and dementia remained significant after further adjusting for baseline frailty status, social isolation, depressive symptoms, medication burden, family history of dementia, and *APOE* ε4 carrier (Table S4). In addition, excluding participants with addiction history or those developing dementia in the first two years of follow-up did not materially change the relationship between computer gaming and dementia (Table S4).

After adjusting for baseline processing speed (reaction time; *N* = 468,122) and visuospatial memory (pairs matching; *N* = 467,744), we found that high frequency of playing computer games was significantly associated with a decreased risk of dementia (Table S5). However, due to the relatively small sample size with limited statistical power, the effect of computer gaming on dementia did not reach significance after adjusting for baseline prospective memory (*N* = 155,619), verbal and numerical reasoning (fluid intelligence; *N* = 152,166), and attention/working memory (numeric memory; *N* = 47,737) (Table S5).

### Associations of computer gaming with cognition and brain structure

Compared with participants who never played computer games, those who sometimes played computer games (prospective memory: OR, 1.30 [95% CI: 1.22, 1.39]; reaction time: beta, -0.086 [95% CI: -0.108, -0.064]; fluid intelligence: beta, 0.183 [95% CI: 0.160, 0.205]; numeric memory: beta, 0.064 [95% CI: 0.037, 0.091]; pairs matching: beta, -0.208 [95% CI: -0.231, -0.186]) and those who often played computer games (prospective memory: OR, 1.46 [95% CI: 1.26, 1.70]; reaction time: beta, -0.195 [95% CI: -0.243, -0.147]; fluid intelligence: beta, 0.334 [95% CI: 0.286, 0.382]; numeric memory: beta, 0.107 [95% CI: 0.047, 0.166]; pairs matching: beta, -0.253 [95% CI: -0.302, -0.203]) had better performance in cognitive assessments (all *P* for trend < 0.001) (Table 3). In addition, high frequency of playing computer games was associated with high volume of gray matter in hippocampus (beta, 0.078; 95% CI: 0.023, 0.134; *P* for trend = 0.006) (Table 3).

**Table 3:** Associations of computer gaming with cognition and brain structure

	Frequency of playing computer games			*P* for trend
	Never/rarely	Sometimes	Often	
**Cognitive functions (** ***N*** ** = 41,267 to 59,184)** ^*^				
Prospective memory (OR [95% CI])	Reference	1.30 (1.22, 1.39)	1.46 (1.26, 1.70)	< 0.001
Reaction time (Beta [95% CI])	Reference	-0.086 (-0.108, -0.064)	-0.195 (-0.243, -0.147)	< 0.001
Fluid intelligence (Beta [95% CI])	Reference	0.183 (0.160, 0.205)	0.334 (0.286, 0.382)	< 0.001
Numeric memory (Beta [95% CI])	Reference	0.064 (0.037, 0.091)	0.107 (0.047, 0.166)	< 0.001
Incorrect pairs matching (Beta [95% CI])	Reference	-0.208 (-0.231, -0.186)	-0.253 (-0.302, -0.203)	< 0.001
**Brain structural measures (** ***N*** ** = 40,059)** ^**†**^				
Volume of gray matter (Beta [95% CI])	Reference	-0.011 (-0.032, 0.010)	-0.025 (-0.070, 0.021)	0.284
Volume of white matter (Beta [95% CI])	Reference	0.032 (0.005, 0.059)	-0.036 (-0.094, 0.022)	0.226
Total brain volume (Beta [95% CI])	Reference	0.010 (-0.013, 0.034)	-0.036 (-0.087 0.014)	0.155
Volume of white matter hyperintensities (Beta [95% CI])	Reference	0.043 (0.018, 0.069)	0.044 (-0.011, 0.100)	0.118
Volume of hippocampus (Beta [95% CI])	Reference	-0.006 (-0.032, 0.020)	0.041 (-0.016, 0.098)	0.157
Volume of gray matter in hippocampus (Beta [95% CI])	Reference	-0.012 (-0.037, 0.014)	0.078 (0.023, 0.134)	0.006

^*^ All expressed as z score units (standardized mean difference), except prospective memory which was expressed as an odds ratio (OR)

^†^ All expressed as z score units (standardized mean difference)

These analyses were adjusted for sex, age, age-square, smoking status, alcohol drinking, educational attainment, Townsend deprivation index, employment status, diet score, sedentary duration, physical activity, sleep duration, loneliness, history of diabetes, and history of cardiovascular disease

Abbreviation: CI, confidence interval

### MR analyses

In the MR study, a total of 68 SNPs were included as genetic instruments for the frequency of playing computer games (Figure S2). The phenotypic variance of the frequency of playing computer games explained by the genetic instruments was 0.59%. The F-statistic for the genetic instruments of the frequency of playing computer games is 40, suggesting that there is no weak instrument bias in this MR study [33].

**Fig. 2:**
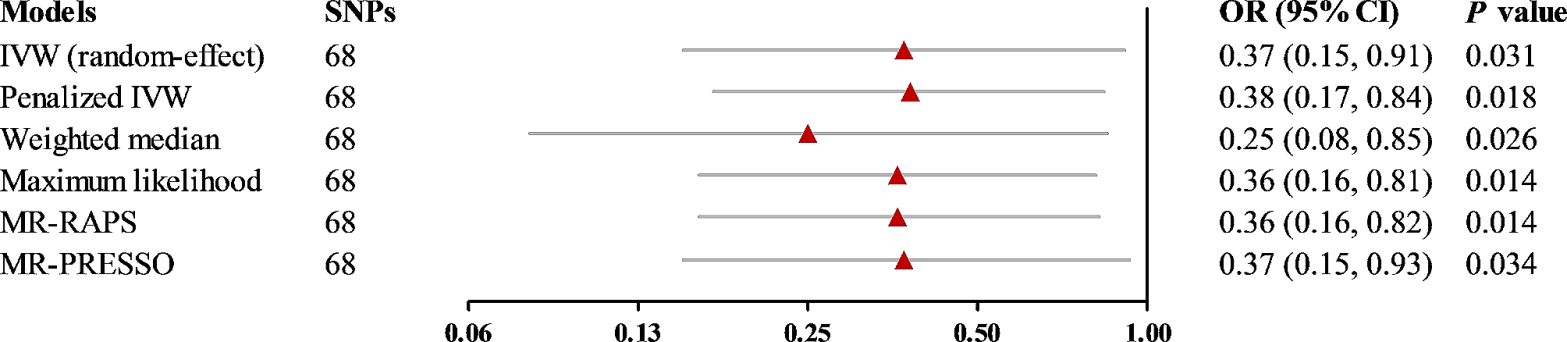
Forest plots for the association between genetically predicted frequency of playing computer games and the risk of dementia. Effect estimates were derived from the main Mendelian randomization (MR) analysis (inverse-variance weighted [IVW] method) and a series of sensitivity analyses (the penalized IVW method, the weighted median method, the maximum likelihood method, the MR-Robust Adjusted Profile Scoring [MR-RAPS] method, and the MR Pleiotropy Residual Sum and Outlier [MR-PRESSO] method). Abbreviations: CI, confidence interval; OR, odds ratio; SNP, single nucleotide polymorphism

In the main IVW MR analysis, genetically determined high frequency of playing computer games was associated with a decreased risk of dementia (OR, 0.37; 95% CI: 0.15, 0.91; *P* = 0.031) (Fig. 2). Associations of each genetic instrument for the frequency of playing computer games with the risk of dementia are presented in Figure S2. In the sensitivity analyses with penalized IVW MR method, the weighted median method, the maximum likelihood method, the MR-RAPS method, and the MR-PRESSO method, the association between genetically determined frequency of playing computer games and incident dementia remained significant (Fig. 2). The MR-PRESSO global test and the MR-Egger regression indicated that there was no pleiotropy for the identified association (Table S6). In addition, the leave-one-out analysis showed that no individual SNP substantially drove the identified association of computer gaming with the risk of dementia (Figure S2). The multivariable MR analyses adjusting for hypertension, diabetes, hyperlipidemia, smoking, and alcohol drinking did not reveal any attenuation of the identified association (Table S7).

## Discussion

To our knowledge, this is the first study to comprehensively assess the associations of computer gaming with incident dementia, cognitive functions, and brain structure. In the prospective study with 471,346 White British participants based on the UK Biobank, we found that higher frequency of playing computer games was associated with lower risk of dementia. Subgroup analyses and sensitivity analyses further confirmed the association between computer gaming and dementia. Moreover, our prospective analyses also suggested significant associations of high frequency of playing computer games with better cognitive functions and brain structural measures. In the two-sample MR analysis with 172,517 European-descent participants, we found that there was a potential causal association between computer gaming and decreased risk of dementia.

Previous epidemiologic studies have suggested protective effects of active video games on cognitive function [5–11]. For example, in a cross-sectional analysis based on the 503 participants from Amazon Mechanical Turk, video gamers were observed to perform better on verbal working memory, visuospatial working memory, and n-back task than non-gamers [6]. Ballesteros et al. conducted a RCT among 40 Spanish older adults and found that training with non-action video games could improve the processing speed, attention, and immediate and delayed visual recognition memory of the participants [7]. In a meta-analysis of 18 RCTs with 1023 older adults, virtual reality exergames could provide potential positive influences on cognition and memory [8]. Similarly, another meta-analysis of 9 RCTs with 409 mild cognitive impairment/dementia patients revealed a favorable effect of video gaming interventions on Mini-Mental State Examination score, suggesting that video gaming had therapeutic potential for mild cognitive impairment and dementia [11].

In the present prospective study with a larger sample size, compared with participants who never played computer games, those who often played computer games had a 19% decreased risk of incident dementia. We also found that high frequency of playing computer games was associated with better performance in multiple cognitive domains, including prospective memory, processing speed, verbal and numerical reasoning, attention/working memory, and visuospatial memory. Moreover, despite the promising role of computer gaming in retarding cognitive impairment, causality for the association of computer gaming with incident dementia has not been previously assessed. In the present large-scale MR study, we further demonstrated a significant association between genetically determined high frequency of playing computer games and decreased risk of dementia.

Brain structural changes resulting from computer gaming are widely implicated in the pathogenesis of dementia [3, 4]. It has been reported that individuals training with 3D-platform games displayed growth in the hippocampus or the functionally connected entorhinal cortex [3]. Similar improvement in brain structure was observed for individuals receiving dance video game training, with significantly increased prefrontal cortex activity after training [4]. Our findings extended this information to gray matter in hippocampus, and revealed the potential association between computer gaming and high volume of gray matter in hippocampus. There are several possible mechanisms underlying the low risk of dementia in individuals with high frequency of playing computer games. Previous studies suggested that video gaming could activate the sympathetic nervous system [34], while sympathetic activation was deemed as a critical contributor to enhancing cognitive states [35]. Moreover, video gaming was shown to be able to attenuate high-fat meal-induced endothelial dysfunction [36]. Of note, vascular endothelial homeostasis was involved in normal functioning of the brain, such as the management of cerebral blood flow, the regulation of inflammatory and immune responses, and the secretion of some bioactive molecules (e.g., brain-derived neurotrophic factor) [37]. On the other hand, as well-known pathophysiological bases in the development of dementia, the improvement of cardiovascular health and emotional condition in response to active video games was even greater than to traditional exercises [38]. Furthermore, it has been reported that action video games could foster brain plasticity by enhancing attentional control and processing speed [39]. In addition, emerging evidence showed that video-game training was capable of enriching cognitive stimulation in older adults and in turn to counteract the aging-related decline of default mode network functional connectivity [40]. Therefore, based on these potential mechanisms, computer gaming may influence the cognitive function and the development of dementia. Further studies are warranted to investigate the detailed mechanisms underlying the protective effects of computer gaming on dementia.

The incidence of dementia remains stubbornly high, so effective and tractable targets for dementia prevention are needed to reduce the burden of dementia by aggressive monitoring and early intervention. In this large-scale prospective study based on the UK Biobank, we found that computer gaming was associated with improved cognitive functions and decreased risks of dementia, suggesting that computer gaming might be a potential preventive target for dementia. However, the effects of video game training on cognitive function are influenced by game types and game devices [41], and some action video games may even increase participants’ sensitivity to aggression signals [42]. Therefore, further clinical trials are warranted to explore the favorable habits of computer gaming and their risk-modifying effects on dementia.

Our study has some strengths. The prospective study was based on the UK Biobank with large sample size, which enabled us to perform analyses with a high statistical power. In addition to assessing the observational association between computer gaming and dementia, this is the first MR study to examine the potential causality for the association between computer gaming and dementia. The well-designed large-scale GWASs and the multiple MR methods ensured that our study could provide a valid appraisal of the causality for the association between computer gaming and dementia. Certain limitations of this study should also be discussed here. Firstly, data on the frequency changes of playing computer games over time were not available, so we were unable to investigate the association between dynamic changes of the frequency of playing computer games and incident dementia. Secondly, given that a big sample size enables us to find statistically significant differences between groups with small absolute differences, our findings concerning the relatively small beneficial effects of computer gaming on cognitive functions may not be directly interpreted as clinical benefits of computer gaming for cognition. Thirdly, the dementia cases in our study were diagnosed during hospital admissions. Therefore, the milder dementia cases who might not seek hospital treatments were likely to be underreported, which may introduce potential misclassification biases in this study. Fourthly, MR estimates are susceptible to the influences of invalid instruments and pleiotropy. However, in the present study, sensitivity analyses with different MR models yielded similar results as the main analyses, and the MR-PRESSO global test and MR-Egger regression suggested no pleiotropy for the identified association. Therefore, the possibility of invalid instrument and pleiotropy was minimal. Finally, all the participants were from Europe, which limited the generalizability of our findings. Further studies conducted among other populations with different ethnic background are needed to confirm our findings.

## Conclusions

In this comprehensive study, we found that high frequency of playing computer games was associated with decreased risk of dementia, favorable cognitive functions, and better brain structure. In addition, MR analysis further showed a potential causal association between computer gaming and dementia. These findings suggest that computer gaming could modulate cognitive function and may be a valuable preventive target for dementia.

### Electronic supplementary material

Below is the link to the electronic supplementary material.

Supplementary Material 1

## Data Availability

Data from UK Biobank (https://www.ukbiobank.ac.uk/) and FinnGen (https://Www.Finngen.Fi) are available to researchers on application. Part of this research has been conducted using the UK Biobank Resource under Application 91185. Statistical code is available on the request by directly contacting the corresponding author (email: zbzhu@suda.edu.cn).

## Abbreviations

CI: Confidence interval
GWAS: Genome-wide association study
HR: Hazard ratio
IVW: Inverse-variance weighted
MR: Mendelian randomization
MRI: Magnetic resonance imaging
MR-PRESSO: MR Pleiotropy Residual Sum and Outlier
MR-RAPS: MR-Robust Adjusted Profile Scoring
OR: Odds ratio
RCT: Randomized controlled trial
SNP: Single nucleotide polymorphism
