# Supplementary material for: Associations of computer gaming with incident dementia, cognitive functions, and brain structure: a prospective cohort study and Mendelian randomization analysis
Source: Alzheimers Res Ther. 2024 Jun 19;16:131. doi: 10.1186/s13195-024-01496-7 (PMC11186151; doi:10.1186/s13195-024-01496-7)
Supplement: Supplementary file 1 — Supplementary Material 1 [file 13195_2024_1496_MOESM1_ESM.docx]

**Supplementary materials**

**Associations of computer gaming with incident dementia, cognitive functions, and brain structure: a prospective cohort study and Mendelian randomization analysis**

**Supplementary Methods**

**MR study design**

We examined the potential causal association between the frequency of playing computer games and dementia via Mendelian randomization (MR) design (Figure 1)^12^. Summary statistics utilized in the present study were obtained from European ancestry-based genome-wide association studies (GWASs)^14-16^. The protocol and data collection were approved by the ethics committee of the original GWASs, and written informed consent was obtained from each participant before data collection.

**Data sources**

Summary-level data on the frequency of playing computer games was derived from a large-scale GWAS based on the UK Biobank, involving 462433 European-descent participants^14, 15^. UK Biobank is a prospective cohort study that included over 500000 men and women from the UK general population between 2006 and 2010. We accessed the data through the MRC IEU OpenGWAS website: <https://gwas.mrcieu.ac.uk/datasets/ukb-b-4779/>.

Summary-level data on dementia was derived from the FinnGen study^16^. This is a nationwide Finnish GWAS meta-analysis of 13 cohorts and biobanks, including 5933 cases and 166584 controls of European ancestry. Dementia cases were defined by ICD-10 code F00-F03, F051, and G30; ICD-9 code 290, 2912A, 2828C, 2941A, 3310A, 3311A, and 4378A; and ICD-8 code 290. We accessed the data through the MRC IEU OpenGWAS website: <https://gwas.mrcieu.ac.uk/datasets/finn-b-KRA_PSY_DEMENTIA_EXMORE/>.

**Genetic instruments for the frequency of playing computer games**

In this MR study, we selected single nucleotide polymorphisms (SNPs) associated with the frequency of playing computer games at genome-wide significance (*P* value <5×10^-8^) and being not in linkage disequilibrium (LD) with other SNPs (r^2^ <0.01 within a clumping window of 400 kb) as instruments to proxy for the frequency of playing computer games. When we encountered SNPs above the LD threshold of r^2^ =0.01, only the SNPs with the lowest *P* value for association with the frequency of playing computer games was selected. SNPs which were not available in dementia dataset were further excluded. Accordingly, a total of 68 independent SNPs for the frequency of playing computer games were retained in the present MR study (Figure S2). Subsequently, we calculated the phenotypic variance of the frequency of playing computer games explained by the genetic instruments via the package gtx in R (version 3.4.3; R Development Core Team). Furthermore, we computed the F-statistic to estimate the strength of the genetic instruments for the frequency of playing computer games^33^. An F-statistic greater than 10 suggested a strong instrument.

**Statistical analysis**

In the main analysis, the random-effect inverse-variance weighted (IVW) method was applied to examine the association between genetically determined frequency of playing computer games and the risk of dementia^26^. However, the IVW estimates may be biased in the presence of invalid instruments or pleiotropy, so we further performed a series of sensitivity analyses with different MR models to assess the robustness of the primary findings. Among the sensitivity analyses, the penalized IVW method would penalize the SNPs with pleiotropic effects^27^; the weighted median method could provide reliable estimates when <50% of genetic instruments were invalid^28^; the maximum likelihood method could make valid causal inference in the presence of measurement error in SNP-exposure association^14^; the MR-Robust Adjusted Profile Scoring (MR-RAPS) method was robust to the violation of the key MR assumptions^29^; the MR Pleiotropy Residual Sum and Outlier (MR-PRESSO) method could detect the extent of horizontal pleiotropy by the MR-PRESSO global test, and the estimates derived from this method were based on outlier correction^30^; the MR-Egger regression method reflected the directional pleiotropy via the intercept term^31^; the leave-one-out method could determine whether an individual SNP significantly drives the studied association by leaving each of them out of MR analysis in turn^32^. Finally, we utilized multivariable MR models with adjustment for hypertension, diabetes, hyperlipidemia, smoking, and alcohol drinking to explore the relatively direct associations between the frequency of playing computer games and dementia^31^. Summary statistics for these confounders were derived from the GWAS datasets based on the UK Biobank, involving 54358 hypertension cases and 408652 controls (available from IEU GWAS database: <https://gwas.mrcieu.ac.uk/datasets/ukb-b-12493/>), 22340 diabetes cases and 439238 controls (available from IEU GWAS database: <https://gwas.mrcieu.ac.uk/datasets/ukb-b-10753/>), 3439 hyperlipidemia cases and 459571 controls (available from IEU GWAS database: <https://gwas.mrcieu.ac.uk/datasets/ukb-b-17462/>), 462434 samples for smoking (available from IEU GWAS database: <https://gwas.mrcieu.ac.uk/datasets/ukb-b-223/>), and 462346 samples for alcohol drinking (available from IEU GWAS database: <https://gwas.mrcieu.ac.uk/datasets/ukb-b-5779/>).

The results are presented as odds ratios (ORs) and their 95% confidence intervals (CIs) of dementia. All statistical analyses were performed in R software (version 3.4.3; R Development Core Team) with ‘gtx’, ‘MendelianRandomization’, ‘MRPRESSO’, and ‘TwoSampleMR’ packages.

**Table S1. Codes used in the UK Biobank to identify incident dementia.**

| **ICD-9** | **ICD-10** |
| --- | --- |
| 290.2, 290.3, 290.4, 291.2, 294.1, 331.0, 331.1, 331.2, 331.5 | A81.0, F00, F00.0, F00.1, F00.2, F00.9, F01, F01.0, F01.1, F01.2, F01.3, F01.8, F01.9, F02, F02.0, F02.1, F02.2, F02.3, F02.4, F02.8, F03, F05.1, F10.6, G30, G30.0, G30.1, G30.8, G30.9, G31.0, G31.1, G31.2, G31.8, G31.9, I67.3 |

**Table S2. Description of cognitive tests.**

| **Cognitive test** | **Questions/measures** | **Domains** | **Ranges** | **Definition of better performance** |
| --- | --- | --- | --- | --- |
| Prospective memory | “At the end of the games we will show you four coloured shapes and ask you to touch the Blue Square. However, to test your memory, we want you to actually touch the Orange Circle instead." | Prospective memory | 0 versus 1 | 1 = correct at first attempt |
| Reaction time | Mean time to correctly identify matches of two cards (unit: millisecond) | Processing speed | 150-1809 | Faster reaction (reverse coded) |
| Fluid intelligence | 2 min to complete as many questions as possible; number of correctly answered items out of 13 questions | Verbal and numerical reasoning | 0-13 | Higher fluid intelligence score |
| Numeric memory | Memorize 2-digit number; maximum digits remembered correctly | Attention/working memory | 2-12 | More digits remembered correctly |
| Pairs matching | Match 3 pairs of 6 cards in 5 seconds in the first round and match 6 pairs of 12 cards in 5 seconds in the second round; number incorrect matches/errors | Visuospatial memory | 0-48 | More correct match (reverse coded) |

**Table S3. Subgroup analysis of hazard ratios (95% CI) of incident dementia according to the frequency of playing computer games.**

| **Subgroup** | **Cases/participants (%)** | **Frequency of playing computer games** | | | ***P* for trend** | ***P* for interaction** |
| --- | --- | --- | --- | --- | --- | --- |
|  |  | **Never/rarely** | **Sometimes** | **Often** |  |  |
| Age | | | | | | 0.091 |
| <60 years | 1185/262134 (0.5) | 1.00 (reference) | 0.88 (0.73, 1.05) | 0.96 (0.68, 1.35) | 0.280 |  |
| ≥60 years | 6713/209212 (3.2) | 1.00 (reference) | 0.90 (0.82, 0.98) | 0.77 (0.64, 0.91) | <0.001 |  |
| Smoking status | | | | | | 0.332 |
| Current | 3505/253422 (1.4) | 1.00 (reference) | 0.90 (0.79, 1.01) | 0.81 (0.62, 1.06) | 0.023 |  |
| Past | 3396/167015 (2.0) | 1.00 (reference) | 0.93 (0.83, 1.04) | 0.88 (0.70, 1.09) | 0.107 |  |
| Never | 946/49267 (1.9) | 1.00 (reference) | 0.78 (0.62, 0.98) | 0.62 (0.40, 0.96) | 0.004 |  |
| With college degree | | | | | | 0.824 |
| No | 6069/313963 (1.9) | 1.00 (reference) | 0.85 (0.72, 1.00) | 0.95 (0.70, 1.29) | 0.134 |  |
| Yes | 1567/149706 (1.1) | 1.00 (reference) | 0.91 (0.83, 0.99) | 0.76 (0.63, 0.92) | <0.001 |  |
| Daily sleep duration | | | | | | 0.596 |
| 6 hours or less | 2096/112721 (1.9) | 1.00 (reference) | 0.85 (0.73, 0.99) | 0.84 (0.63, 1.12) | 0.033 |  |
| 7 or 8 hours | 4704/319943 (1.5) | 1.00 (reference) | 0.94 (0.85, 1.03) | 0.80 (0.65, 0.99) | 0.023 |  |
| 9 hours or more | 989/36097 (2.7) | 1.00 (reference) | 0.80 (0.63, 1.01) | 0.74 (0.47, 1.18) | 0.034 |  |
| Feeling lonely | | | | | | 0.521 |
| No | 6763/429670 (1.6) | 1.00 (reference) | 0.90 (0.83, 0.98) | 0.81 (0.69, 0.96) | <0.001 |  |
| Yes | 514/21501 (2.4) | 1.00 (reference) | 0.80 (0.59, 1.09) | 0.73 (0.42, 1.28) | 0.097 |  |

In the multivariable models, sex, age, age-square, smoking status, alcohol drinking, educational attainment, Townsend deprivation index, employment status, diet score, sedentary duration, physical activity, sleep duration, loneliness, history of diabetes, and history of cardiovascular disease were included unless the variable was used as a subgroup variable.

Abbreviation: CI: confidence interval.

**Table S4. Sensitivity analyses for the association between computer gaming and the risk of dementia.**

| **Frequency of playing computer games** | | | ***P* for trend** |
| --- | --- | --- | --- |
| **Never/rarely** | **Sometimes** | **Often** |  |
| **Further adjusted for baseline frailty status (N = 442590)** | | | |
| 1.00 (reference) | 0.90 (0.83, 0.98) | 0.81 (0.69, 0.95) | <0.001 |
| **Further adjusted for baseline social isolation (N = 461908)** | | | |
| 1.00 (reference) | 0.90 (0.83, 0.97) | 0.82 (0.70, 0.97) | <0.001 |
| **Further adjusted for baseline depressive symptoms (N = 442355)** | | | |
| 1.00 (reference) | 0.91 (0.84, 0.98) | 0.82 (0.70, 0.97) | 0.001 |
| **Further adjusted for baseline medication burden (N = 471008)** | | | |
| 1.00 (reference) | 0.90 (0.83, 0.97) | 0.81 (0.69, 0.95) | <0.001 |
| **Further adjusted for family history of dementia (N = 462275)** | | | |
| 1.00 (reference) | 0.90 (0.83, 0.97) | 0.79 (0.67, 0.93) | <0.001 |
| **Further adjusted for *APOE* ε4 carrier (N = 385578)** | | | |
| 1.00 (reference) | 0.90 (0.82, 0.98) | 0.82 (0.68, 0.98) | 0.001 |
| **Excluding participants with addiction history (N = 462328)** | | | |
| 1.00 (reference) | 0.90 (0.83, 0.97) | 0.80 (0.68, 0.94) | <0.001 |
| **Excluding dementia cases occurring in the first two years of follow-up (N = 471194)** | | | |
| 1.00 (reference) | 0.90 (0.84, 0.98) | 0.81 (0.69, 0.95) | <0.001 |

The analyses were based on Model 2 in the main analysis (adjusted for sex, age, age-square, smoking status, alcohol drinking, educational attainment, Townsend deprivation index, employment status, diet score, sedentary duration, physical activity, sleep duration, loneliness, history of diabetes, and history of cardiovascular disease).

**Table S5. Association between computer gaming and dementia after adjustment for baseline cognitive functions.**

| **Frequency of playing computer games** | | | ***P* for trend** |
| --- | --- | --- | --- |
| **Never/rarely** | **Sometimes** | **Often** |  |
| **Further adjusted for baseline prospective memory (N = 155619)** | | | |
| 1.00 (reference) | 1.03 (0.89, 1.19) | 1.11 (0.84, 1.47) | 0.456 |
| **Further adjusted for baseline processing speed (reaction time; N = 468122)** | | | |
| 1.00 (reference) | 0.94 (0.87, 1.01) | 0.85 (0.72, 0.99) | 0.012 |
| **Further adjusted for baseline verbal and numerical reasoning (fluid intelligence; N = 152166)** | | | |
| 1.00 (reference) | 1.06 (0.92, 1.22) | 1.19 (0.90, 1.58) | 0.184 |
| **Further adjusted for baseline attention/working memory (numeric memory; N = 47737)** | | | |
| 1.00 (reference) | 1.14 (0.89, 1.46) | 1.44 (0.90, 2.30) | 0.087 |
| **Further adjusted for family visuospatial memory (incorrect pairs matching; N = 467744)** | | | |
| 1.00 (reference) | 0.92 (0.85, 1.00) | 0.84 (0.72, 0.98) | 0.004 |

The analyses were based on Model 2 in the main analysis (adjusted for sex, age, age-square, smoking status, alcohol drinking, educational attainment, Townsend deprivation index, employment status, diet score, sedentary duration, physical activity, sleep duration, loneliness, history of diabetes, and history of cardiovascular disease).

**Table S6. Pleiotropy assessment for the MR findings.**

| **SNPs** | **MR-PRESSO global test** | | **MR-Egger intercept** | | |
| --- | --- | --- | --- | --- | --- |
|  | **Observed RSS** | ***P* value** | **Beta** | **SE** | ***P* value** |
| 68 | 87.58 | 0.066 | 0.032 | 0.016 | 0.056 |

Abbreviations: MR, Mendelian randomization; MR-PRESSO, MR pleiotropy residual sum and outlier; RSS, residual sum of square; SE, standard error; SNP, single nucleotide polymorphism.

**Table S7. Multivariable MR analyses of the association between genetically determined frequency of playing computer games and the risk of dementia.**

| **SNPs** | **OR (95% CI)** | ***P* value** |
| --- | --- | --- |
| **Adjusted for hypertension (ukb-b-12493; N = 463010)^*^** | | |
| 68 | 0.34 (0.14, 0.85) | 0.021 |
| **Adjusted for diabetes (ukb-b-10753; N = 461578)^*^** | | |
| 68 | 0.33 (0.13, 0.89) | 0.028 |
| **Adjusted for hyperlipidemia (ukb-b-17462; N = 463010)^*^** | | |
| 65 | 0.34 (0.13, 0.86) | 0.023 |
| **Adjusted for smoking (ukb-b-223; N = 462434)^*^** | | |
| 68 | 0.30 (0.12, 0.74) | 0.009 |
| **Adjusted for alcohol drinking (ukb-b-5779; N = 462346)^*^** | | |
| 68 | 0.35 (0.14, 0.89) | 0.027 |

^*^ We accessed the data through the MR-Base platform (UKB-b: X) to obtain the GWAS datasets on these confounders based on UK Biobank samples (<https://gwas.mrcieu.ac.uk/>).

Abbreviations: CI, confidence interval; GWAS, genome-wide association study; MR, Mendelian randomization; OR, odds ratio; SNP, single nucleotide polymorphism.


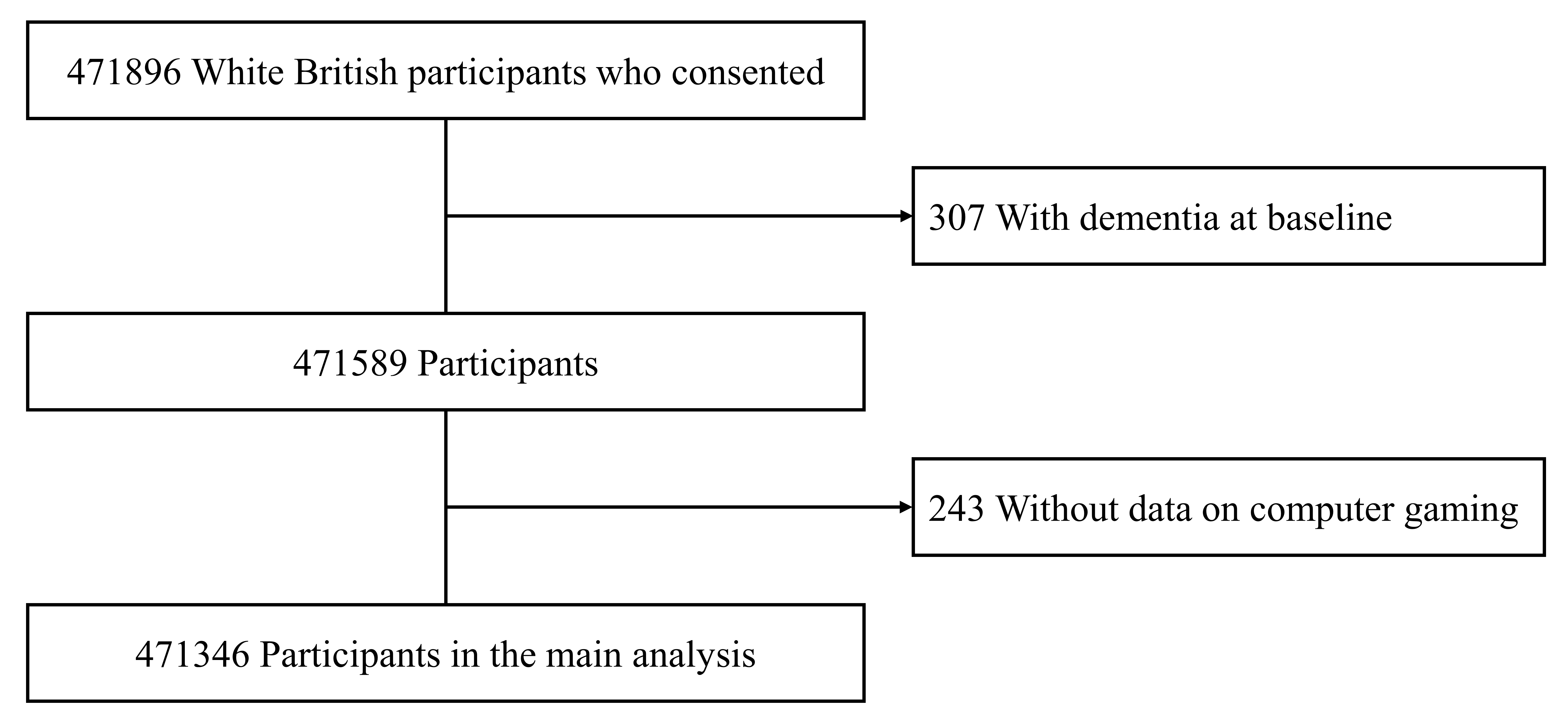


**Figure S1. Flow diagram of participant selection.**





**Figure S2. Association of computer gaming with the risk of dementia.**

(A) scatter plot of genetic associations with computer gaming and dementia and (B) forest plot of leave-one-out analysis for computer gaming on dementia. Odds ratios (ORs) and confidence intervals (CIs) are represented per unit change in the frequency of playing computer games.

Abbreviation: SNP, single nucleotide polymorphism.
